# Optimization of therapeutic strategies for selective lateral lymph node dissection after neoadjuvant chemoradiotherapy in patients with rectal cancer with clinical suspected lateral lymph node metastasis

**DOI:** 10.3389/fonc.2023.1271463

**Published:** 2023-10-10

**Authors:** Yuan Liu, Mandoula Bao, Yujuan Jiang, Feng Li, Wei Xing, Zhufeng Yang, Qian Liu

**Affiliations:** ^1^ Department of Surgery, Hebei Province Hospital of Chinese Medicine/Affiliated Hospital of Hebei University of Chinese Medicine, Shijiazhuang, China; ^2^ Department of Colorectal Surgery, National Cancer Center/National Clinical Research Center for Cancer/Cancer Hospital, Chinese Academy of Medical Sciences and Peking Union Medical College, Beijing, China; ^3^ Department of General Surgery, Beijing Friendship Hospital, Capital Medical University, Beijing Key Laboratory of Cancer Invasion and Metastasis Research and National Clinical Research Center of Digestive Diseases, Beijing, China

**Keywords:** safety, surgical indications, survival outcomes, lateral lymph node dissection, neoadjuvant chemoradiotherapy

## Abstract

**Background:**

Selective lateral lymph node (LLN) dissection with total mesorectal excision after neoadjuvant chemoradiotherapy (nCRT) is pointed out to reduce lateral compartment recurrence and to improve survival in patients with rectal cancer with LLN metastases. This study aimed to explore the safety, surgical indications, and survival outcomes of LLN dissection after nCRT.

**Methods:**

This multicenter retrospective study included patients with rectal cancer with clinical evidence of LLN metastases (n = 466) treated across three hospitals in China. Patients who underwent total mesorectal excision and LLN dissection were grouped into nCRT (n = 155) and non-nCRT (n = 291), respectively. Propensity score matching was used to minimize selection bias.

**Results:**

After matching, nCRT did not significantly increase the surgery duration, intraoperative blood loss or postoperative complications (*P* > 0.05). In a multivariate logistic regression analysis, poor/mucinous/signet adenocarcinoma (*P* = 0.042) and post-nCRT LLN short diameter ≥7 mm (*P* < 0.001) were independent risk factors for pathological LLN metastasis after nCRT. Overall survival (*P* < 0.001) and disease-free survival (*P* < 0.001) were significantly worse in patients with LLN metastasis, which was, however, not an independent risk factor for survival after eliminating confounders. Multivariate prognostic analysis of 40-patient subset with pathological LLN metastasis showed that distant metastasis, metastasis beyond the obturator or internal iliac region, and ≥2 LLN metastasis were independent predictors of poor overall survival.

**Conclusions:**

Selective LLN dissection after nCRT is safe and feasible with acceptable perioperative outcomes. Patients with a post-nCRT LLN short diameter ≥7 mm or poor/mucinous/signet adenocarcinoma should receive supplementary LLN dissection after nCRT. However, patients with distant metastasis, metastasis beyond the obturator or internal iliac region, and involvement of ≥2 LLN may not benefit from LLN dissection, and LLN dissection should be carefully considered in such patients.

## Introduction

Lateral lymph nodes (LLNs) are one of the important lymphatic drainage areas for middle-low rectal cancer (1). Two therapeutic methods for LLN metastasis, LLN dissection (LLND) and neoadjuvant chemoradiotherapy (nCRT), have been topics of debate worldwide (2, 3). Recent literature revealed that neither LLND alone nor nCRT can completely eradicate metastatic LLN, and both have relatively high risk of lateral compartment recurrence (4, 5).

In recent years, surgeons in Eastern and Western countries have recommended selective LLND after nCRT for patients with clinically evidence of LLN metastasis, which can improve local control while avoiding unnecessary LLND, thus reducing overtreatment and morbidity (6, 7). Although LLN short-diameter–based indications for LLND after nCRT have been reported previously, the cutoff values vary widely among literature (8–12). In addition, patients with LLN metastases are often associated with systemic metastases, and the survival benefit is not promising even when LLND is performed (11, 13). Therefore, we performed a multicenter retrospective study to evaluate the safety, surgical indication, and prognosis for LLND after nCRT. In addition, we also aim to investigate the patient characteristics that could indicate benefits from LLND in cases of pathological LLN metastasis, which could optimize the therapeutic strategies of LLND.

## Patients and methods

### Patient

This is a multicenter retrospective study using data from a prospectively collected institutional database from three institutions. Patients with rectal cancer with clinical suspected LLN metastasis who underwent total mesorectal excision (TME) with LLND from January 2011 to December 2019 were identified.

The inclusion criteria were as follows: (1) age ranges from 18–75 years old; (2) middle-low rectal cancer; and (3) histopathology-confirmed adenocarcinoma, mucinous adenocarcinoma, or signet-ring cell carcinoma. The exclusion criteria were as follows: (1) clinical T1–T2; (2) simultaneous distant metastases that cannot achieve curative resection; and (3) history of other malignant tumors. Finally, a total of 446 patients were included in this study (198 cases from the Cancer Hospital, Chinese Academy of Medical Sciences, and Peking Union Medical College; 162 cases from the Peking University First Hospital; and 86 cases from Peking Union Medical College). The study was approved by the institutional review boards of the three hospitals, and the study design was registered (NCT04850027) at ClinicalTrials. gov.

### Diagnosis and treatment strategy

The LLN status and clinical TNM stage before and after nCRT were assessed by two radiologists who specialized in gastrointestinal diseases, and the LLN diameter was measured with electronic calipers on pelvic magnetic resonance imaging (MRI). Patients with clinical LLN metastases were all treated with LLND, and clinically suspected LLN metastasis was diagnosed by meeting any of the following diagnostic criteria: (1) short-axis diameter ≥0.5 cm before nCRT; (2) inhomogeneous or intense enhancement; or (3) irregular shape and rough edges. TNM staging was performed according to the American Joint Committee on Cancer staging system (eighth edition) (14). Therapeutic strategies were determined by multidisciplinary team meetings including radiologists and medical and surgical physicians and ultimately depend on the patient’s wishes. The enrolled patients were divided into nCRT or non-nCRT groups according to the preoperative treatment strategy. Patients in the nCRT group received a long course of chemoradiotherapy (50.4 Gy in 25 fractions with concurrent capecitabine) to include the coverage of the LLN basin. Pelvic MRI was performed 6 weeks after nCRT completion to re-evaluate the radiologic characteristic of the swollen LLN and record it in detail. TME with LLND were performed 6–8 weeks after completion of nCRT. The detailed procedure of LLND has been described previously (15). The extent of LLND was in accordance with the Japanese Society for Cancer of the Colon and Rectum guidelines, including four regions: common iliac vessels, internal iliac vessels, external iliac vessels, and obturator area (2). Severe complications were defined as grade IIIa complications and greater according to the Clavien–Dindo classification (16).

### Follow-up

Follow-up examinations was conducted according to the national comprehensive cancer network (NCCN) guidelines through outpatient; physical examination and serum tumor marker evaluations [carcinoma embryonic antigen (CEA) and carbohydrate antigen (CA) 19-9] were performed every 3 months; and CT examinations of the chest, abdomen, and pelvis were performed every 6 months for the first 3 years. Three years after surgery, the patients were followed up every 6–12 months until death or 31 November 2021. Oncological outcomes included overall survival (OS; the time from the primary surgery to death) and disease-free survival (DFS; the time from the primary surgery to recurrence or metastasis). Recurrence or metastasis was confirmed by histopathology, typical radiographic evidence, or an elevated carcinoembryonic antigen level. Alive and recurrence-free patients were censored at every follow-up, and data were collected based on a follow-up survey.

### Statistical analysis

Data analysis were conducted by Statistical Package for the Social Sciences (SPSS) version 24.0 (IBM Corp., Armonk, NY, USA) in this study. To reduce the imbalance between the two groups, propensity score matching (PSM) was conducted to match patients in the nCRT group 1:1 with those in the non-nCRT group (caliper = 0.1), and the covariates included gender, age, American Society of Anesthesiologists (ASA) score, body mass index (BMI), distance from anal verge (AV), surgical approach, operative type, LLND, clinical TNM stage, and LLN short diameter.

Quantitative data were expressed as mean ± standard deviation and analyzed using the paired *t-*test or Mann–Whitney U-test. Categorical variables were compared using the chi-square test or the Fisher’s exact test and presented as frequencies and percentages. Univariate logistic regression analysis was performed to explore the association between clinical variables and LLN metastasis. Clinical variables with P < 0.05 in univariate analysis were included in the multivariate logistic regression model to examine the independent predictors of LLN metastasis. The Kaplan–Meier method and log-rank test were used to calculate and compare the OS and DFS. The statistically significant variables in univariate analysis were subsequently tested by multivariate analysis using a Cox regression model, and the hazard ratio (HR) with a 95% confidence interval (CI) was used to assess the effect of each variable. Statistical significance was set at *P* < 0.05.

## Results

### Baseline data and clinical characteristics

**Figure 1:**
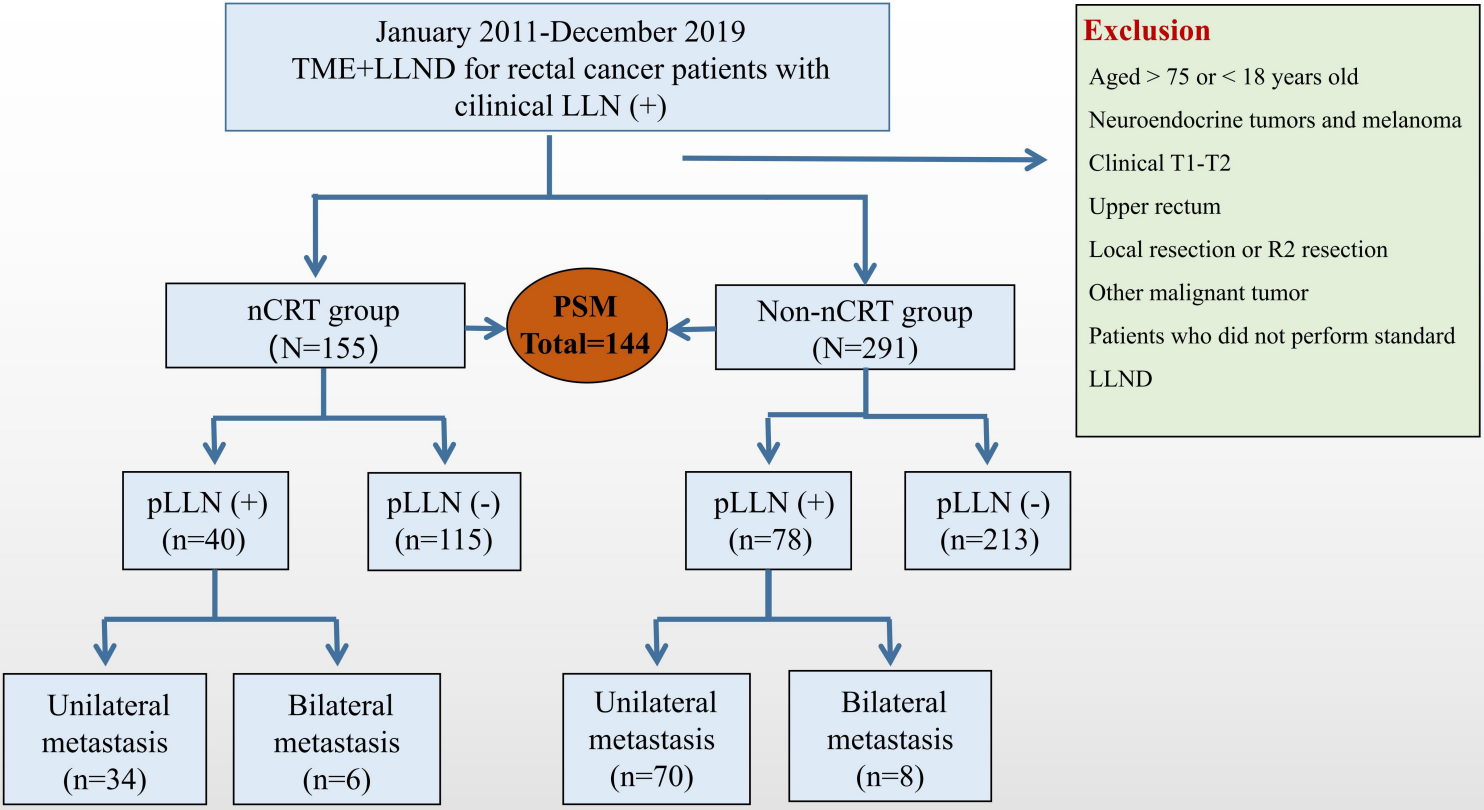
Research flowcharts. LLN, lateral lymph node; TME, total mesorectal excision; nCRT, neoadjuvant chemoradiotherapy; LLND, lateral lymph node dissection; PSM, propensity score-matched.

A total of 446 patients who underwent TME + LLND were eligible and were enrolled in present study. Among them, 155 patients received nCRT and 291 patients underwent upfront surgery without nCRT. Seventy-two matched pairs were selected through PSM and stratified into the nCRT group (n = 72) and non-nCRT group (n = 72) (**Figure 1**).

The baseline data and clinical characteristics before and after PSM between the groups are shown in **Table 1**. Before PSM, compared with the non-nCRT group, patients in the nCRT group had lower tumor locations (4.1 vs. 4.9 cm, *P* = 0.014), and more patients underwent laparoscopic LLND (89.0% vs. 59.8%, *P* < 0.001). In addition, the proportion of patients with clinical stage III (83.2% vs. 57.4%, *P* < 0.001) and LLN short diameter ≥10 mm (49.0% vs. 32.3%, *P* < 0.001) was higher in the nCRT group. After PSM, the nCRT and non-nCRT groups were well balanced in terms of the above variables.

**Table 1:** The baseline data and clinical characteristics before and after PSM between group.

Variables	Total cohort			Matched cohort		
	nCRT group (n = 155)	Non-nCRT (n = 291)	*P*	nCRT group (n = 72)	Non-nCRT (n = 72)	*P*
Gender (%)			0.362			0.611
Male	99 (63.9)	173 (59.5)		44 (61.1)	41 (56.9)	
Female	56 (36.1)	118 (40.5)		28 (38.9)	31 (43.1)	
Age at operation (years, mean ± SD)	55.7 ± 11.2	57.9 ± 11.7	0.057	56.2 ± 8.4	57.1 ± 8.8	0.293
BMI (kg/m^2^, mean ± SD)	24.4 ± 3.4	23.8 ± 3.3	0.476	24.3 ± 2.8	23.6 ± 3.2	0.485
ASA score (%)			0.651			1.000
I–II	146 (94.2)	277 (95.2)		69 (95.8)	69 (95.8)	
III	9 (5.8)	14 (4.8)		3 (4.2)	3 (4.2)	
Distance from AV (cm, mean ± SD)	4.1 ± 2.0	4.9 ± 3.1	0.014	4.2 ± 2.0	4.4 ± 2.5	0.522
Surgical approach			<0.001			0.229
Open	17 (11.0)	117 (40.2)		13 (18.1)	19 (26.4)	
Laparoscopic	138 (89.0)	174 (59.8)		59 (81.9)	53 (73.6)	
Operative type			0.260			0.815
Low anterior resection	70 (45.2)	140 (48.1)		32 (44.4)	36 (50.0)	
Abdominoperineal resection	61 (39.4)	121 (41.6)		29 (40.3)	28 (38.9)	
Hartmann procedure	9 (5.8)	16 (5.5)		3 (4.2)	3 (4.2)	
Total pelvic exenteration	15 (9.6)	14 (4.8)		8 (11.1)	5 (6.9)	
LLN dissection			0.351			0.594
Unilateral dissection	109 (70.3)	192 (66.0)		50 (69.4)	47 (65.3)	
Bilateral dissection	46 (29.7)	99 (34.0)		22 (30.6)	25 (34.7)	
Clinical TNM stage			<0.001			0.369
II	26 (16.8)	124 (42.6)		20 (27.8)	25 (34.7)	
III	129 (83.2)	167 (57.4)		52 (72.2)	47 (65.3)	
LLN short diameter			0.001			0.611
≥10 mm	76 (49.0)	94 (32.3)		31 (43.1)	28 (38.9)	
<10 mm	79 (51.0)	197 (67.7)		41 (56.9)	44 (61.1)	

BMI, body mass index; ASA, American Society of Anesthesiologists; AV, anal verge; LLN, lateral lymph node; PSM, propensity score matching.

### Perioperative data and pathological results

The perioperative data and pathological results are summarized in **Table 2**. After PSM, compared with the non-nCRT group, the nCRT group had longer operation time (*P* = 0.174) and more estimated blood loss (*P* = 0.433), but the difference was not statistically significant. There was no significant difference in postoperative complications and total hospital stay between the two groups (*P* > 0.05). The proportion of patients with pathological T3–T4 (69.4% vs. 86.1%, *P* < 0.001) was lower in the nCRT group. In addition, nCRT significantly reduced the number of mesorectal lymph node (LN) dissections (13.6 vs. 15.9, *P* = 0.035).

**Table 2:** Perioperative data and pathological outcomes before and after PSM between group.

Variables	Total cohort			Matched cohort		
	nCRT group (n = 155)	Non-nCRT (n = 291)	*P*	nCRT group (n = 72)	Non-nCRT (n = 72)	*P*
Operation time (min, mean ± SD)	300.7 ± 103.6	277.4 ± 111.2	0.018	289.2 ± 82.3	268.3 ± 90.2	0.174
Estimated blood loss (ml, mean ± SD)	134.1 ± 114.4	121.4 ± 107.8	0.355	129.4 ± 119.4	110.3 ± 112.3	0.433
Postoperative complications (%)	31 (20.0)	48 (16.5)	0.356	16 (22.2)	14 (19.4)	0.682
Bowel obstruction	4 (2.6)	9 (3.1)		2 (2.8)	3 (4.2)	
Abdominal or perineal incision infection	3 (1.9)	7 (2.4)		2 (2.8)	2 (2.8)	
Pelvic abscess	8 (5.2)	15 (5.2)		3 (4.2)	2 (2.8)	
Anastomotic leakage	5 (3.2)	10 (3.4)		3 (4.2)	3 (4.2)	
Anastomotic hemorrhage	1 (0.6)	2 (0.7)		0 (0)	0 (0)	
Pelvic hemorrhage	1 (0.6)	3 (1.0)		0 (0)	0 (0)	
Obturator nerve damage	3 (1.9)	6 (2.1)		1 (1.4)	2 (2.8)	
Urinary retention	13 (8.4)	22 (7.6)		4 (5.6)	5 (6.9)	
Respiratory infections	6 (3.9)	8 (2.7)		2 (2.8)	1 (1.4)	
Chylous ascites	3 (1.9)	5 (1.7)		2 (2.8)	1 (1.4)	
Other	0 (0)	3 (1.0)		0 (0)	0 (0)	
Total hospital stay (day, mean ± SD)	12.8 ± 10.6	12.1 ± 10.3	0.761	12.4 ± 9.3	11.8 ± 8.9	0.826
Mortality (%)	1 (0.6)	1 (0.2)	1.000	1 (1.4)	0 (0)	1.000
Pathological T stage (%)			0.010			0.016
Complete response or T1–T2	46 (29.7)	55 (18.9)		22 (30.6)	10 (13.9)	
T3–T4	109 (60.3)	236 (81.1)		50 (69.4)	62 (86.1)	
Pathological mesorectal LN metastasis (%)			0.007			0.182
N0	65 (41.9)	161 (55.3)		40 (55.6)	32 (44.4)	
N1–N2	90 (58.1)	130 (44.7)		32 (44.4)	40 (55.6)	
Histology (%)			0.083			0.567
Moderate	122 (78.7)	207 (71.1)		55 (76.4)	52 (72.2)	
Poor/Mucinous/signet	33 (21.3)	84 (28.8)		17 (23.6)	20 (27.8)	
Pathological LLN metastasis (%)			0.820			0.257
Yes	40 (25.8)	78 (26.8)		16 (22.2)	22 (30.6)	
No	115 (74.2)	213 (73.2)		56 (77.8)	50 (69.4)	
Bilateral LLN metastasis (%)	6 (3.9)	8 (2.7)	0.717	3 (4.2)	1 (1.4)	0.620
LLNs removed (n, mean ± SD)	9.0 ± 6.9	8.9 ± 6.2	0.860	9.2 ± 6.6	8.8 ± 6.0	0.872
Mesorectal LN removed (n, mean ± SD)	13.8 ± 7.8	16.3 ± 9.3	0.004	13.6 ± 7.4	15.9 ± 8.0	0.035
Adjuvant therapy			<0.001			0.580
Yes	121 (78.1)	175 (60.1)		50 (69.4)	53 (73.6)	
No	34 (21.9)	116 (39.9)		22 (30.6)	19 (26.4)	

LLN, lateral lymph node; LN, lymph node; PSM, propensity score matching.

### Short diameter distributions of LLN before and after nCRT

**Figure 2:**
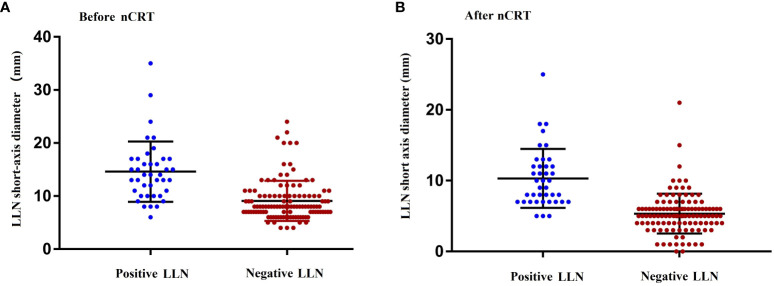
Maximum short diameter of the LLNs as determined by MRI before **(A)** and after **(B)** nCRT. nCRT, neoadjuvant chemoradiotherapy; LLN, later lymph node; MRI, magnetic resonance imaging.

**Figure 2** shows the distribution of the LLN short-axis diameter before and after nCRT. In comparison with LLN-negative patients, LLN-positive patients showed larger mean short-axis diameters of the LLN before (*P* < 0.001) and after nCRT (*P* = 0.007).

The negative predictive value, positive predictive value, specificity, sensitivity, and area under the curve (AUC) of a pre-nCRT short-axis diameter of 10 mm for predicting pathological LLN metastasis were 92.8%, 47.2%, 66.9%, 85.0%, and 0.760, respectively, whereas the corresponding values for a post-nCRT cutoff LLN short-axis diameter of 7 mm were 96.8%, 59.7%, 78.2%, 92.5%, and 0.884, respectively (**Table 3**). Hence, LLN short-axis diameters of 10 mm and 7 mm before and after nCRT, respectively, were determined to be used as the cutoff values for predicting LLN metastasis in this study.

**Table 3:** Diagnosis of LLN metastasis by short diameter.

Time lines	Cutoff value	Negative predictive value	Positive predictive value	Specificity	Sensitivity	AUC
Pre-nCRT LLN short diameter						
	7 mm	96.0%	27.9%	20.9%	97.5%	0.592
	8 mm	97.8%	35.8%	39.1%	97.5%	0.683
	9 mm	94.5%	43.9%	60.0%	90.0%	0.750
	10 mm	92.8%	47.2%	66.9%	85.0%	0.760
Post-nCRT LLN short diameter						
	5 mm	100%	34.8%	34.8%	100%	0.674
	6 mm	95.5%	42.0%	55.6%	92.5%	0.741
	7 mm	96.8%	59.7%	78.2%	92.5%	0.884
	8 mm	89.1%	62.2%	85.2%	70.0%	0.776

LLN, lateral lymph node.

### Predictive preoperative variables for LLN metastasis after nCRT

Univariate and multivariate analyses of the predictive preoperative variables for LLN metastasis are summarized in **Table 4**. Univariate analysis reveals that a pre-nCRT LLN short-axis diameter ≥10 mm (*P* < 0.001), distant metastasis (*P* = 0.013), poor/mucinous/signet adenocarcinoma (*P* < 0.001), and post-nCRT LLN short-axis diameter ≥7 mm (*P* < 0.001) were associated with an increased possibility of LLN metastasis. Multivariate analysis demonstrated that mucinous/signet-ring adenocarcinoma [odd ratio (OR) =3.100; 95% CI, 1.040–9.238; P = 0.042] and post-nCRT LLN short-axis diameter ≥7 mm (OR = 22.767; 95% CI, 5.885–88.078; *P* < 0.001) were independent predictive variables for pathological LLN metastasis.

**Table 4:** Predictive preoperative factors for LLN metastasis after nCRT.

Variables	Univariable analysis			Multivariable analysis		
	Positive LLN (n = 40)	Negative LLN (n = 115)	*P*	OR	95% CI	*P*
Gender			0.187			
Male	29 (72.5)	70 (60.9)				
Female	11 (27.5)	45 (39.1)				
Age at operation	55.1 ± 13.1	55.9 ± 10.5	0.746			
Distance from AV	4.2 ± 2.5	4.4 ± 1.8	0.704			
Tumor size	4.1 ± 2.2	3.4 ± 1.9	0.079			
Pre-nCRT CEA level	13.3 ± 27.3	11.5 ± 22.5	0.694			
Pre-nCRT CA 19-9 level	44.3 ± 86.0	22.1 ± 46.1	0.138			
Pre-nCRT LLN short diameter			<0.001	2.808	0.814–9.690	0.102
<10 mm	6 (15.0)	77 (70.0)				
≥10 mm	34 (85.0)	38 (30.0)				
Distant metastasis			0.013	2.694	0.596–12.176	0.918
Presence	8 (20.0)	6 (5.2)				
Absence	32 (80.0)	109 (94.8)				
cT stage			0.923			
cT1–T2	4 (10.0)	9 (7.8)				
cT3–T4	36 (90.0)	106 (92.2)				
cN stage (mesorectal LN)			0.155			
cN0	3 (7.5)	21 (18.2)				
cN1–N2	33 (92.5)	94 (81.8)				
Histologic grade			<0.001	3.100	1.040–9.238	0.042
Moderate	23 (57.5)	99 (86.1)				
Poor/Mucinous/signet	17 (42.5)	16 (13.9)				
Post-nCRT CEA level	3.0 ± 3.0	3.9 ± 4.8	0.428			
Post-nCRT CA 19-9 level	25.2 ± 63.6	14.1 ± 9.4	0.456			
Post-nCRT LLN short diameter			<0.001	22.767	5.885–88.078	<0.001
<7 mm	3 (7.5)	90 (78.3)				
≥7 mm	37 (92.5)	25 (21.7)				
LLN location			0.650			
Unilateral	27 (67.5)	82 (71.3)				
Bilateral	13 (32.5)	33 (28.7)				

AV, anal verge; nCRT, neoadjuvant chemoradiotherapy; LLN, lateral lymph node; LN, lymph node.

### Prognosis of TME with LLND after nCRT

**Figure 3:**
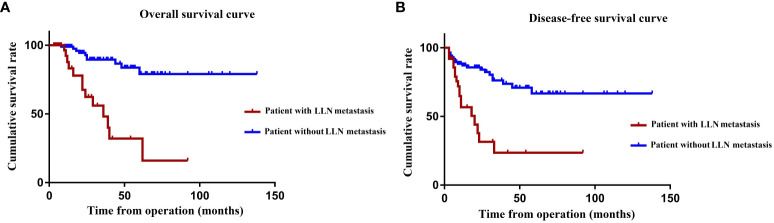
OS **(A)** and DFS **(B)** according to the presence of LLN metastasis. OS, overall survival; DFS, disease-free survival; LLN, lateral lymph node.

During the mean follow-up period of 40.2 months, 29 of the 155 patients died, whereas 47 patients presented local recurrence or distant metastasis. The OS (*P* < 0.001) and DFS (*P* < 0.001) after TME + LLND combined with nCRT in patients with pathological LLN metastasis were significantly lower than those in patients without pathological LLN metastasis. The 3- and 5-year OS rates in patients with pathological LLN metastasis were 48.5% and 32.3%, respectively, and those in patients without metastasis were 89.5% and 79.1%, respectively (**Figure 3A**). The 3- and 5-year DFS rates were 23.6% and 23.6%, respectively, in patients with pathological LLN metastasis, and 76.3% and 66.7%, respectively, in patients without pathological LLN metastasis (**Figure 3B**). However, multivariate analysis demonstrated that OS was significantly affected by lymphatic invasion (HR, 10.35; 95% CI, 2.15–49.91; *P* = 0.004) and N2 stage (HR, 6.57; 95% CI, 1.20–35.92; *P* = 0.030). DFS was also significantly affected by lymphatic invasion (HR, 3.48; 95% CI, 1.21–9.99; *P* = 0.021) and distant metastasis (HR, 32.95; 95% CI, 7.82–138.81; *P* < 0.001) (**Table 5**).

**Table 5:** Univariate and multivariate regression analyses for prognosis of TME with LLND after nCRT.

Variables	Overall survival				Disease-free survival			
	Univariate analysis		Multivariate analysis		Univariate analysis		Multivariate analysis	
	HR (95% CI)	*P*	HR (95% CI)	*P*	HR (95% CI)	P	HR (95% CI)	*P*
Gender: male/female	0.95 (0.41–2.20)	0.904			1.09 (0.56–2.12)	0.810		
Age at operation	1.01 (0.97–1.05)	0.761			1.01 (0.98–1.04)	0.636		
ASA score: I–II/III	1.19 (0.16–8.84)	0.865			2.01 (0.28–14.64)	0.492		
Pre-nCRT CEA level	1.00 (0.99–1.02)	0.657			1.01 (1.00–1.03)	0.013	1.01 (0.99–1.02)	0.223
Pre-nCRT CA 19-9 level	1.01 (0.99–1.01)	0.031	1.00 (0.99–1.01)	0.851	1.00 (1.00–1.01)	0.015	1.00 (0.99–1.01)	0.281
Distant metastasis	5.89 (2.31–15.04)	<0.001	1.57 (0.32–7.80)	0.582	43.92 (14.24–135.49)	<0.001	32.95 (7.82–138.81)	<0.001
Histology (Poor, Mucinous or signet/moderate)	3.41 (1.19–9.77)	0.023	1.53 (.039–6.05)	0.541	2.42 (1.07–5.48)	0.034	3.04 (0.99–9.31)	0.052
Operative type: laparoscopic/open	0.80 (0.29–2.18)	0.656			0.91 (0.38–2.18)	0.825		
Post-nCRT CEA level	1.02 (0.93–1.13)	0.649			1.01 (0.93–1.10)	0.902		
Post-nCRT CA 19-9 level	1.01 (0.99–1.02)	0.082			1.01 (0.99–1.01)	0.209		
Lymphatic invasion (yes/no)	15.44 (4.52–52.76)	<0.001	10.35 (2.15–49.91)	0.004	6.12 (2.64–14.20)	<0.001	3.48 (1.21–9.99)	0.021
Perineural invasion (yes/no)	2.86 (1.08–7.54)	0.034	1.67 (0.50–5.57)	0.402	1.92 (0.89–4.17)	0.098		
Vascular invasion (yes/no)	2.49 (0.87–7.13)	0.089			1.87 (0.80–4.41)	0.150		
Pathological T stage (T3–T4/T1–T2)	4.19 (1.24–14.12)	0.021	3.02 (0.55–7.80)	0.201	2.14 (0.98–4.67)	0.058		
Pathological N stage (mesorectal LN)								
N0	–	–			–	–		
N1	5.74 (1.55–21.29)	0.009	3.31 (0.57–19.22)	0.183	3.05 (1.37–6.80)	0.006	1.16 (0.40–3.35)	0.790
N2	17.63 (4.85–64.10)	<0.001	6.57 (1.20–35.92)	0.030	5.39 (2.35–12.37)	<0.001	0.89 (0.23–3.42)	0.868
Pathological LLN metastasis (yes/no)	6.39 (2.77–14.75)	<0.001	1.28 (0.35–4.65)	0.712	3.99 (2.06–7.74)	<0.001	1.54 (0.54–4.44)	0.422
Postoperative complication (yes/no)	0.97 (0.29–3.27)	0.961			0.82 (0.32–2.10)	0.675		
Grade 3–5 postoperative complication (no/yes)	2.65 (0.62–11.40)	0.191			1.18 (0.28–4.89)	0.822		

LLN, lateral lymph node; TME, total mesorectal excision; nCRT, neoadjuvant chemoradiotherapy; LLND, lateral lymph node dissection; LN, lymph node.

### Prognosis for patients with pathological LLN metastasis

We performed univariate and multivariate analyses to investigate the prognostic factors for OS in patients with pathological LLN metastasis. Distant metastasis (HR, 9.43; 95% CI, 1.96–45.35; *P* = 0.005), metastasis beyond the obturator or internal iliac region (HR, 4.77; 95% CI, 1.16–19.56; *P* = 0.030), and more than one LLN metastasis (HR, 5.12; 95% CI, 1.21–21.75; *P* = 0.027) were independent predictive factors for OS in multivariate analysis (**Table 6**).

**Table 6:** Univariate and multivariate regression analyses for overall survival of patients with LLN metastasis after nCRT.

Variables	Overall survival			
	Univariate analysis		Multivariate analysis	
	HR (95% CI)	P	HR (95% CI)	P
Gender: male/female	0.70 (0.22–2.21)	0.541		
Age at operation	0.98 (0.94–1.03)	0.442		
Pre-nCRT CEA level	0.99 (0.96–1.02)	0.487		
Pre-nCRT CA 19-9 level	1.00 (0.99–1.00)	0.996		
Distant metastasis	3.99 (1.14–13.96)	0.030	9.43 (1.96–45.35)	0.005
Histology (Poor, Mucinous or signet/moderate)	0.79 (0.16–3.96)	0.776		
nCRT and surgery interval (≥6 weeks/<6 weeks)	0.25 (0.47–1.28)	0.096		
Post-nCRT CEA level	1.06 (0.85–1.31)	0.618		
Post-nCRT CA 19-9 level	1.00 (0.99–1.01)	0.408		
Pre-nCRT LLN size (≥10 mm/<10 mm)	0.97 (0.20–4.69)	0.968		
Post-CRT LLN size (≥7 mm/<7 mm)	0.65 (0.75–5.67)	0.698		
Pathological T stage (T3–T4/T1–T2)	0.52 (0.10–2.57)	0.419		
Pathological N stage (mesorectal LN)				
N0	–	–		
N1	0.69 (0.12–4.00)	0.680		
N2	2.90 (0.49–17.08)	0.240		
LLN metastasis location (obturator or internal iliac/other)	6.11 (1.62–23.12)	0.008	4.77 (1.16–19.56)	0.030
Number of LLN metastasis (≥2/< 2)	4.04 (1.21–13.50)	0.024	5.12 (1.21–21.75)	0.027

LLN, lateral lymph node; nCRT, neoadjuvant chemoradiotherapy.

## Discussion

There are various therapeutic strategies to treat the LLN metastasis, such as the bilateral LLND (5), selective LLND only for swollen LN detected on preoperative MRI (8–12), and omitting LLND and replacing it with nCRT (17). The optimal treatment strategy has not yet been developed in Eastern and Western countries. In China, surgeons perform selective LLND after nCRT on the basis of radiological features to avoid overtreatment while enhancing local control in the lateral compartment. Therefore, the purpose of this study was to evaluate the safety, indications, and prognostic analysis of TME with LLND after nCRT.

The safety and feasibility of LLND after nCRT have always been a concern of surgeons. Our study showed that patients who received nCRT showed longer operation times (289.2 vs. 268.3min, *P* = 0.174) and more estimated blood loss (110.3 vs. 129.4 mL, *P* = 0.433) for LLND than those who did not performed nCRT in matched cohort, but the difference was not statistically significant. In addition, the incidence of postoperative complications was similar in both groups (22.2% vs. 19.4%, *P* = 0.682). Therefore, we point out that LLND is safe and feasible after nCRT in experienced institutions.

The short diameter of the LLN is significantly relate to local recurrence (18), and whether LLND should be performed after nCRT is determined by the surgeon and radiologist on the basis of the short diameter, edge, and heterogeneity of the LLN as assessed by MRI. However, the cutoff values for LLN short diameter vary widely among literature (8–12). Our previous study revealed that patients with an LLN short-axis diameter ≥7 mm after nCRT or undesirable histological type are supposed to receive supplementary LLND after nCRT (15). Similarly, Inoue et al. reported that 7 mm could be a more appropriate cutoff value for post-nCRT LLN short diameter (12). The present study also showed that post-nCRT LLN short diameter ≥7 mm and poor/mucinous/signet-ring adenocarcinoma were predictive risk factors for pathological LLN metastasis. We point out that, if our criteria of an LLN short diameter ≥7 mm were used in present study, then 92.5% of cases with LLN metastasis can be identified and helped avoid LLND in 58.1% of the cases.

In the present study, the OS and DFS after nCRT combined with LLND in patients with pathological LLN metastasis were significantly worse than those in patients without pathological LLN metastasis; these differences could be attributed to different stage distributions, because 20% of the patients with pathological LLN metastasis had stage IV disease, and only 5.2% of those without pathological LLN metastasis had stage IV disease. To balance the interference of confounding factors on oncological outcomes, we conducted a multivariate analysis, and the results showed that pathological LLN metastasis was not an independent risk factor affecting OS and DFS. Therefore, we suggest that LLN metastasis is a regional LN metastasis with a good survival outcomes after nCRT combined with LLND and that dissection is valuable if necessary. However, we also found some patients with LLN metastasis survived for more than 5 years after LLND, whereas others relapsed or died only 6 months. Therefore, to further optimize the surgical indications for LLND after nCRT, we conducted a prognostic analysis of 40 patients with pathological LLN metastasis to explore the patient characteristics that may indicate prognostic benefit from LLND. Previous literature has reported that the actual number and region of metastatic LLN have a significant adverse impact on prognosis (19, 20). Similarly, our study also found that patients with liver metastasis, metastasis beyond the obturator or internal iliac region, and those with more than one LLN metastasis might not benefit from LLND after nCRT; therefore, the decision to perform LLND for these patients should be made with caution.

The treatment of LLN metastasis is essentially a multidisciplinary and comprehensive treatment. In this study, 9.0% (14/155) of patients in the nCRT group had distant metastases at the time of initial diagnosis. We suggest that enhanced adjuvant chemotherapy is needed to improve the elimination of micro-metastases. However, as a complex technique, LLND has a high risk of complications and poor compliance to constrain the administration of postoperative adjuvant chemotherapy. Under the premise of controlling the toxicity and side effects, supplementary chemotherapy before or after radiotherapy (totally neoadjuvant therapy) may be considered or might even replace radiotherapy with strengthening chemotherapy (21, 22).

Indeed, nCRT should be recommended for all patients with rectal cancer with T3 and N+ according to the NCCN guidelines. However, 291 patients in this study did not receive nCRT. The reason for the low proportion of nCRT in present study is that the study spans 8 years and the therapeutic strategies are constantly improving and being optimized. In the early years, the concept of nCRT for rectal cancer was rarely applied. In recent years, the concept of nCRT has been gradually promoted and applied. For patients with a high risk of relapse such as T4 stage, multiple LN metastases, nCRT were considered and selected. Furthermore, therapeutic strategy for LLN metastases was updated during the study period. Between 2011 and 2017, patients with clinical LLN metastasis mainly performed upfront surgery without nCRT. After 2018, nCRT should be performed before LLND for patients with LLN short diameter ≥10 mm. However, nCRT is recommended by physician and ultimately requires the patient’s consent to be performed. For family or economic reasons, even some patients who meet the indications still refuse nCRT and request surgery first, which resulted in a low proportion of nCRT in this study.

There are several limitations to this study. First, the sample size was relatively small, with only 446 patients included. However, unlike in Japan, prophylactic LLND is not routinely adopted in China. Hence, we suggest that this study could be conducted as a large-scale multicenter series in China. Second, the prognostic analysis in this study showed that the location and actual number of metastatic LLN were poor prognostic factors. The decision to perform LLND is based on preoperative examination, but these prognostic factors were obtained from pathological findings that were unavailable to the physician before operation. However, with continuous improvement and development in radiological technology, the short diameter, location, quantity, heterogeneity, and other radiologic characteristics of the metastatic LLN can be evaluated accurately by preoperative MRI. Hence, we suggest that the outcomes of this study can still provide reference and guidance for clinical work. Finally, the retrospective nature led to a certain selection bias, such as patient selection, treatment strategy, and data integrity. In the future, a multi-center randomized controlled study will be conducted to further confirm our conclusions and provide better evidence-based medical evidence for the diagnosis and treatment of LLN metastasis.

## Conclusions

Selective LLND after nCRT is safe and feasible with acceptable perioperative outcomes. Patients with a post-nCRT LLN short diameter ≥7 mm or poor/mucinous/signet adenocarcinoma should receive supplementary LLND after nCRT. However, LLND should be carefully considered in patients with distant metastases, metastases beyond the obturator or internal iliac region, and multiple-LLN involvement.

## Data availability statement

The raw data supporting the conclusions of this article will be made available by the authors, without undue reservation.

## Ethics statement

All the procedures followed the ethical standards of the World Medical Association Declaration of Helsinki. The study was approved by the institutional review boards of the three hospitals, and the study design was registered (NCT04850027) at ClinicalTrials. gov. The studies were conducted in accordance with the local legislation and institutional requirements. The participants provided their written informed consent to participate in this study.

## Author contributions

YL: Conceptualization, Data curation, Formal Analysis, Methodology, Writing – original draft, Writing – review & editing. MB: Conceptualization. YJ: Data curation, Formal Analysis, Methodology, Writing – review & editing. FL: Methodology, Writing – review & editing. WX: Data curation, Methodology, Writing – review & editing. ZY: Data curation, Formal Analysis, Project administration, Supervision, Validation, Writing – original draft, Writing – review & editing. QL: Funding acquisition, Project administration, Resources, Supervision, Visualization, Writing – review & editing.
